# Developing evolution-resistant drugs for COVID-19

**DOI:** 10.7554/eLife.81334

**Published:** 2022-07-26

**Authors:** Daniel M Weinreich

**Affiliations:** 1 Department of Ecology, Evolution and Organismal Biology, Brown University Providence United States; 2 Center for Computational Molecular Biology, Brown University Providence United States

**Keywords:** Mpro, SARS-CoV-2, deep mutational scanning, main protease, *S. cerevisiae*

## Abstract

Analyzing how mutations affect the main protease of SARS-CoV-2 may help researchers develop drugs that are effective against current and future variants of the virus.

**Related research article** Flynn JM, Samant N, Schneider-Nachum G, Bakan DT, Yilmaz NK, Schiffer CA, Moquin SA, Dovala D, Bolon DNA. 2022. Comprehensive fitness landscape of SARS-CoV-2 M^pro^ reveals insights into viral resistance mechanisms. *eLife* **11**:e77433. doi: 10.7554/elife.77433.

As of mid-July 2022, more than 12 billion doses of vaccines against SARS-CoV-2, the virus responsible for COVID-19, have been administered to over 5 billion individuals (WHO, 2022). However, mutations that allow the virus to evade vaccines are spreading globally, leading to a need for boosters and updated vaccines (Callaway, 2021; Vogel, 2022). This immunity ‘arms race’ illustrates how efforts to control the COVID-19 pandemic could benefit from additional pharmaceutical approaches.

One of these approaches is the use of inhibitor drugs to block the action of viral proteases, the enzymes that cleave polyproteins encoded in the viral genome to yield several functional proteins. If these proteases are inactivated, the virus cannot synthesize the proteins it requires to reproduce, so inhibiting these enzymes may be an effective way of treating viral diseases. For example, protease inhibitors are key components of highly active antiretroviral therapy (HAART), which renders HIV a chronic disease.

In the case of SARS-CoV-2, many inhibitor candidates have been identified for its main protease, which is called M^pro^ (Jin et al., 2020; Narayanan et al., 2022). One of these inhibitors, with the trade name Paxlovid, has been authorized for emergency use by the United States Federal Drugs Administration (FDA), and pharmacists have been allowed to prescribe it since July 2022 (Owen et al., 2021; FDA, 2022). But can M^pro^ inhibitors be designed to avoid the rapid obsolescence we have observed in vaccines as a result of viral evolution? Now, in eLife, Julia Flynn, Daniel Bolon and colleagues at the University of Massachusetts (UMass) Chan Medical School and the Novartis Institutes for Biomedical Research report the results of experiments assessing the effects of different mutations on the proteolytic activity of M^Pro^ (Flynn et al., 2022).

Using a method called deep mutational scanning (Fowler and Fields, 2014), Flynn et al. generated a ‘library’ that contained almost every single missense mutation of the M^pro^ enzyme relative to the sequence found in the original Wuhan isolate (Wu et al., 2020). Missense mutations are changes to a single nucleotide in the sequence that alter the resulting amino acid. Assessing the activity of the M^Pro^ variants that result from each conceivable missense mutation revealed how they each affect the activity of the enzyme. To do this, Flynn et al. placed the library into yeast cells, and assessed how well each variant of the protease worked in three different environments.

The results showed that the proteolytic activity of each variant was correlated across the three assays, implying that the assays capture the same properties of M^Pro^. Flynn et al. also found a correlation between the results of their assays and previous measurements of the catalytic rates for individual variants. Additionally, the vast majority of the 290 missense mutations of M^Pro^ observed most frequently (at least 100 times) in COVID-19 patients exhibited activity comparable to that of the wild type. This further confirmed that Flynn et al.’s assay captures the biologically relevant function of the enzyme. Finally, the team found that all of the mutation-intolerant residues in M^Pro^ (the sites at which at least 17 out of the 20 alternative amino acids block the protein’s activity) are highly conserved in other coronaviruses. This suggests that inhibitors designed against the current SARS-CoV-2 form of the enzyme will likely be active against future, emergent outbreaks.

Most protease inhibitors act by competitively occupying the enzyme’s binding pocket to the exclusion of its native substrate. Therefore, Flynn et al. decided to further analyze missense mutations in the vicinity of the binding pocket that nevertheless preserve enzyme activity, reasoning that these mutations might represent opportunities for the virus to evolve resistance to inhibitors. Unfortunately, they found that mutations at many of those residues preserve the proteolytic ability of M^pro^ (Cho et al., 2021).

Similarly, and perhaps of more immediate concern, Flynn et al. identified three missense mutations that reduced the binding stability to Paxlovid by at least 1 kcal/mol, corresponding to an approximately five-fold reduction in the drug’s binding affinity, while maintaining proteolytic activity. These represent likely resistance mutations; indeed, a subset of these mutations have now been observed in laboratory populations of SARS-CoV-2 challenged with this version of Paxlovid, as well as in currently circulating isolates (Service, 2022). It will be important to monitor the frequency of these three mutations in patients being treated with Paxlovid as its use becomes more widespread.

But the potential implications of the findings of Flynn et al. go further than providing insight into prospects for resistance evolution against existing M^pro^ inhibitors. Some years ago, the notion of the ‘substrate envelope’ of a viral protease – the overlapping volume occupied by all of its native substrates – was introduced (King et al., 2004). The inspiration was that the substrate specificity of the HIV protease seemed to be determined by the shape of the substrate rather than by a specific amino acid sequence. Because resistance mutations must allow an enzyme to distinguish between its inhibitors and its substrates, they might work by introducing atomic overlaps or disfavored electrostatics in the region where the inhibitor protrudes beyond the substrate envelope. In a recent companion study, researchers at UMass and Novartis (including many authors from Flynn et al.) have reported a high-resolution structure of the SARS-CoV-2 M^pro^ substrate envelope (Shaqra et al., 2022).

Together, these two articles provide clear guidance for the development of evolution-resistant protease inhibitors against SARS-CoV-2. To find clinically durable candidates, compounds should be screened first to identify those that lie entirely within the enzyme’s substrate envelope. Of these, care should be taken to select those that interact with one or more mutation-intolerant residues. This will make it more difficult for the protease to evolve the ability to discriminate between its native substrates and the drug without blocking its activity. Of course, Darwinian evolution is a tremendously powerful force, and resistance has evolved against every antimicrobial class ever deployed in the clinic. Nevertheless, the functional and structural resolution now available for M^pro^-inhibitors offers new optimism 30 months into the COVID-19 pandemic.
